# Epiphytic Bacteria Alter Floral Scent Emissions

**DOI:** 10.1007/s10886-017-0898-9

**Published:** 2017-11-14

**Authors:** Carola Helletsgruber, Stefan Dötterl, Ulrike Ruprecht, Robert R. Junker

**Affiliations:** Department of Ecology and Evolution, University of Salzburg, Hellbrunnerstrasse 34, 5020 Salzburg, Austria

**Keywords:** *Brassica rapa*, Epiphytic bacteria, Floral scent, Microbiome, Volatile organic compounds, Sterile plants

## Abstract

Floral scents are key mediators of biotic interactions between flowers and various organisms such as pollinators, antagonistic animals and bacteria. It has been shown that emissions of floral volatiles are influenced by interactions with other organisms at the levels of roots, leaves and flowers. However, it is largely unknown whether and how epiphytic bacteria associated with flowers affect the composition of floral scent. By comparing volatiles of sterile and inoculated plants we found that bacteria may add components, induce or reduce the emission of compounds, and potentially catabolize others. These mechanisms collectively altered the floral scent emission and led to clearly different compositions. Our results confirm that bacteria have the potential to interfere with flower-animal interactions with consequences for pollination and plant reproduction.

## Introduction

Volatile organic compounds (VOCs) released by flowers are key mediators of biotic interactions with pollinators, antagonistic animals, and bacteria (Junker 2016). These diverse partners do not only behaviorally or physiologically respond to floral scent emissions, they also affect the composition of floral scent, both quantitatively and qualitatively. Whereas herbivore- and pollinator-mediated changes of VOCs have been shown for a number of plant species (Junker 2016), evidence for bacterial effects is limited. A study on *Sambucus nigra* plants showed that flowers after treatment with antibiotics release proportionally altered and reduced emission rates of terpenes, suggesting that bacteria influence floral scent emissions (Penuelas et al. 2014). Several indirect and direct mechanisms by which bacteria may modify floral VOCs are conceivable (Junker and Tholl 2013). First, bacteria induce volatile emissions in plants in response to pathogen attack and avirulent bacterial strains (Huang et al. 2003). Second, bacteria were shown to utilize plant VOCs as carbon source, and their catabolism subsequently adds new compounds to the plants’ scent (Del Giudice et al. 2008). Third, emission rates of volatiles that serve as carbon source for bacteria may strongly decrease as a consequence of bacterial consumption (Abanda-Nkpwatt et al. 2006). Finally, bacteria are known to synthesize and emit a large number of VOCs by their own metabolism independent of plant volatiles that may be used as substrate (Schulz and Dickschat 2007), and these novel compounds may contribute to the plant-specific scent. The mechanisms summarized here have been shown for bacteria associated with roots or leaves, but not for flowers that just recently came into focus as habitats for diverse bacterial colonizers. Given the importance of flowers in the plants’ lifecycle and the growing evidence that bacteria associated with flowers may interfere with pollination (Vannette et al. 2012), which may have consequences for natural and agricultural systems (Aleklett et al. 2014), further information about bacterial effects on floral phenotypes is required.

In order to test the effect of bacteria on floral scent emissions, we compared VOCs of plants cultivated under sterile conditions and of plants cultivated under sterile conditions and inoculated with defined bacterial communities three days prior to floral scent collection. In addition, VOCs of non-sterile greenhouse grown plants were analyzed. Our study contributes to the understanding of how bacteria influence plant traits and provides a basis for future studies, testing the role of bacteria in plant-pollinator interactions and sexual plant reproduction.

## Methods and Materials

### Study Organisms

We used fast-cycling *Brassica rapa* L. plants (Brassicaceae, Wisconsin Fast Plants, 1–033 Standard, Rapid-cycling *Brassica* collection, University of Wisconsin-Madison, Department of Plant Pathology, Madison, USA). Prior to cultivation, seeds were surface-sterilized by treating them with 70% ethanol (2 min) followed by sodium hypochlorite solution (7% available chlorine) containing 0.2% Triton X-100 (8 min), followed by seven washing steps in sterile double-distilled water. Sterilized seeds were cold stratified (4 °C) over night. *B. rapa* plants were grown on a standard Murashige & Skoog (MS) nutrient medium including vitamins (Duchefa Biochemie, Haarlem, Netherlands) supplemented with plant agar (Duchefa Biochemie, Haarlem, Netherlands) and 2% sucrose. After autoclaving the medium, 220 ml were filled into Microboxes (Model: TP5000 + TPD5000–18.5 × 18.5 × 19.1 cm; Combiness nv, Nevele, Belgium) allowing to cultivate one plant individual per box under sterile conditions. Plants were grown under long-day conditions (16 h light – 8 h dark) at a constant temperature of 24 °C. Additional *B. rapa* plants were grown in the green house (non-sterile) under long-day conditions at 20 °C using ED73 standard soil (Einheitserde Werkverband eV, Sinntal-Altengronau, Germany) to evaluate effects of different cultivation methods on scent emission.

Bacterial strains were isolated from *B. rapa* flowers and leaves grown in the Botanical Garden of the University of Salzburg. Individual flowers and leaves were placed in 1 ml autoclaved phosphate buffered saline (PBS tablet, Sigma-Aldrich R, Germany) using sterile forceps and were sonicated for 7 min to detach bacteria from plant surfaces. 50 μL of the PBS containing bacteria were streaked on autoclaved R2A agar medium (Sigma-Aldrich R, Germany) containing cycloheximide (Sigma-Aldrich R, Germany; 30 mg L^−1^) to prevent fungal growth. After an incubation of 72 h, six colony-forming units (cfus, three originating from flowers and three from leaves) were haphazardly selected in order to get an unbiased selection of epiphytic bacteria and were then cultivated on autoclaved LB-medium-powder (Panreac AppliChem, Germany), supplemented with Agar Bacteriology grade (Panreac AppliChem, Germany) and 1 g L^−1^ D-glucose (Sigma-Aldrich R, Germany) without fungicide. Strains were sequenced for identification. The PCR mix contained 0.6 units of GoTaq DNA polymerase (Promega), 0.2 nM of each of the four dNTPs (Promega), 0.3 μm of each primer (Eurofins) and the bacterial cells from the respective colony. The 16S rRNA gene (target regions V3, V4, ~550 bp) was amplified and sequenced (forward and reverse) with two universal primers specific for members of Eubacteria 341f (5′-CCT ACG GGA GGC AGC -3′) and 907rM (5′-CCGTCAATTCMTTTGAGTTT -3′) with standard PCR - conditions (95°C for 2 min: 1 cycle; 94°C for 30 s, 53°C for 30 s, 72°C for 40 s: 35 cycles). Unpurified PCR-products were sent to Eurofins Genomics (Ebersberg, Germany) for sequencing.

Prior to flowering, buds and leaves were inoculated with the mixture of the six selected bacterial strains. Prior to inoculation, each strain was individually suspended in PBS, and initial optical density (OD_600_) was measured using a plate reader (Bio-Tek, BTKELx808iu), and was subsequently diluted to OD_600_ = 0.2. The six suspensions of the bacterial strains were mixed to yield a suspension with even optical densities. Note the same ODs may translate into different concentrations (cell number per volume) for different bacterial strains. Autoclaved cotton swabs were used to inoculate the plants with bacteria. Control plants (sterile) were treated with PBS in the same way.

### VOC Collection and Analyses

VOCs were collected from 19 sterile and eight inoculated plants, as well as from three plants grown under greenhouse conditions by using the dynamic headspace methods as described earlier (Etl et al. 2016). Three days after the inoculation individual plants in full bloom were taken out of the microboxes (including roots and agar) and were bagged in polyethylene oven bags (7 × 10 cm; Toppits, Germany) under sterile conditions (sterile bench). Immediately after bagging, VOCs were trapped for 60 min on adsorbent tubes (quartz glass tubes: length 25 mm; i. diam. 2 mm) filled with 1.5 mg each of Carbotrap B (mesh 20–40) and Tenax TA (mesh 60–80; both Supelco, Germany) using a membrane pump (G12/01 EB; Gardner Denver Thomas GmbH, Fürstenfeldbruck, Germany). The flow was adjusted at 200 ml/min using a flowmeter. For control, headspace samples of empty oven bags and leaves were collected by using the same method. Samples were analyzed with a GC/MS system (QP2010Ultra, Shimadzu Corporation, Japan) coupled to a thermal desorption unit (TD-20, Shimadzu, Japan) and equipped with a ZB-5 fused silica column (5% phenyl, 95% dimethylpolysiloxane; 60 m long, i. diam. 0.25 mm, film thickness 0.25 μm, Phenomenex, USA). We used a split ratio of 1:1 and a consistent helium carrier gas flow of 1.5 ml/min. The GC oven temperature started at 40 °C, then increased by 6 °C/min to 250 °C and was held for 1 min. The MS interface worked at 250 °C. Mass spectra were taken at 70 eV (EI mode) from m/z 30 to 350. GC/MS data were processed using the GCMSolution Version 4.11 (Shimadzu Corporation, Japan). Compounds were identified according to the NIST 11, Wiley 9, FFNSC 2, Essential Oils and Adams 2007 mass spectral (and retention index) databases. The identity of most of the compounds was confirmed by comparison of mass spectra and retention times with those of authentic standards. For quantitative analysis of VOCs, known amounts of monoterpenes, aliphatic, and aromatic compounds were injected into the GC/MS system, and mean peak areas were used to determine the total amount of scent (see Etl et al. 2016). After VOC sampling, bacterial abundances on flowers were checked by isolating bacterial strains from flowers as described above.

### Statistical Analyses

In order to test for differences in VOCs of sterile and inoculated plants, we performed a random forest analysis (*n*_tree_ = 10,000 bootstrap samples with *m*_try_ = 6 compounds randomly selected at each node) using the R package *randomForest*. In addition, we performed a distance-based redundancy analysis as implemented in the R package *vegan* based on Bray-Curtis distances.

## Results and Discussion

Of the six haphazardly selected bacterial strains, three belong to the genus *Staphylococcus* (BR-GH-2A, BR-GH-2B, BR-GH-2C), two to the genus *Bacillus* (BR-GH-3-A, BR-GH-4-A), and one to the genus *Sphingomonas* (BR-GH-4-C, NCBI GenBank accession numbers for nucleotide sequences: MF347688 – MF347693). These bacterial genera have been described to be associated with plant surfaces (Junker and Keller 2015). Bacterial abundance on inoculated flowers ranged from 340,000 to 9,620,000 cfus (mean ± sd: 4,207,500 ± 3,215,881). From 11 out of 19 flowers not inoculated with the bacterial strains, no bacteria were isolated after VOC collection. From the remaining eight flowers, we isolated between 140 and 1200 cfus (mean ± sd: 692.5 ± 425). Contamination most likely occurred during the application of PBS (control treatment) or during VOC collection.

**Fig. 1:**
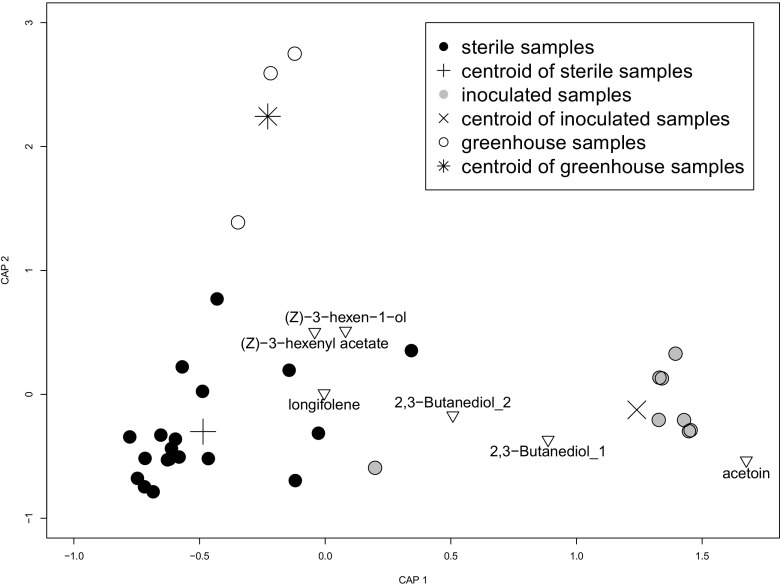
Differences in scent composition of sterile and inoculated *Brassica rapa* inflorescences and of those cultivated in a greenhouse. Similarity of samples returned by distance-based redundancy analysis based on Bray-Curtis distances are shown. Emission rates of compounds in the plot (triangles) are higher in those samples plotted in their direction relative to the origin

Floral scent emissions differed between treatment groups both qualitatively and quantitatively. Sterile, inoculated, and greenhouse plants distinctly differed both in total composition (permutation test for distance-based redundancy analysis: *F*_2,27_ = 5.45, *p* < 0.001, Fig. 1, random forest *class.error*: 0.05 (sterile), 0.13 (inoculated), 0.67 (greenhouse)) and in the emission rates of individual components (Table 1). Most strikingly, acetoin and 2,3-butanediol were detected in considerably higher amounts in samples inoculated with bacteria prior to VOC collection (Table. 1). Exclusively those VOC samples from flowers that were not experimentally inoculated (supposed to be ‘sterile’), but were contaminated with bacteria at the time of VOC collection (eight out of nineteen) contained acetoin and / or its reduction product, 2,3-butanediol, whereas flowers cultivated under sterile conditions from which we did not isolate bacteria after VOC collection were devoid of these compounds. Consequently, bacterial origin of these compounds is suggested, though we cannot exclude that these compounds are plant emissions induced by the bacteria. Both compounds have been described as typical volatiles of bacteria and were shown to be released, among others, by *Staphylococcus* and *Bacillus* (Schulz and Dickschat 2007), supporting the hypothesis that volatiles synthesized by bacteria may well contribute to the chemical phenotype of flowers. In contrast, emission rates of e.g. 1,2-propanediol, 2,3-dimethylpentanol, and longifolene were reduced in samples inoculated with bacteria (Table. 1). Our results strongly suggest that bacteria exploit certain plant VOCs as carbon source, which are, therefore, reduced in their emission rates. Other compounds that occurred exclusively (some of them occasionally) in samples of inoculated flowers (Table. 1) may also represent compounds that are produced directly by bacteria. Alternatively, they might be synthesized by the plants as a response to bacterial colonization, or be breakdown-products of plant VOCs. Samples taken from greenhouse-grown plants, which harbored a more natural bacterial community than the flowers inoculated with our artificial community, also differed from the experimentally treated plants (Fig. 1). These differences may be the result of different cultivation substrates (plant agar vs. soil) and growing conditions and / or differences in bacterial colonization. In the greenhouse-grown flowers we also detected acetoin (Table 1), which corresponds to the presence of bacteria in these samples. While distance-based redundancy analysis and random forest analysis both clearly separated volatile compositions of sterile and inoculated flowers, the former analysis succeeded more in separating the greenhouse samples from the sterile samples than the latter (Fig. 1, Table 1, note the small sample size of greenhouse-grown plants). However, the lack of many compounds in the greenhouse samples as well as the higher emission rates of other compounds (Table 1) demonstrate that sterile plants do emit different bouquets of VOCs than plants with a natural microbiome. Note that the bacterial communities used to inoculate the plant are not as diverse as the communities naturally associated with flowers in diversity and composition. Therefore, future studies should test the effects of natural communities. Nonetheless, our results provide first evidence that epiphytic bacteria have the potential to affect floral scent emissions.

**Table 1:** Scent emission of sterile and inoculated *Brassica rapa* inflorescences and of those cultivated in a greenhouse. For each compound and treatment group mean ± SD emission [ng h^−1^ inflorescence^−1^] is given. Compounds are listed by chemical class and within class sorted by retention index (RI). Variable importance *E* (resulting from random forest analysis) is proportional to the compound’s contribution to a correct assignment. Bold numbers indicate significant differences in the emission rates of these compounds between sterile and inoculated flowers (*t*-test). Compounds marked with asterisk have been identified by using synthetic standards. For compounds with no synthetic standards available, literature data on retention indices (stationary phase: 5% phenyl, 95% dimethylpolysiloxane) are given as footnotes

RI	Compound class and name	greenhouse	sterile	inoculated	*E*
Aliphatic compounds					
684	2-Pentanone*			1.23 ± 1.93	1.34
706	Acetoin*	0.55 ± 0.36	6.18 ± 18.33	409.81 ± 165.93	**49.75**
720	Methyl butyrate*			0.68 ± 1.92	0.00
722^a^	2-Methylbutanenitrile			2.60 ± 6.14	0.90
729^b^	1,2-Propanediol		0.20 ± 0.33		**5.15**
771	2,3-Butanediol_1*		13.49 ± 27.02	226.40 ± 227.99	**32.23**
782	2,3-Butanediol_2*		2.76 ± 6.18	125.05 ± 157.83	32.91
797^c^	2,3-Dimethylpentanol	5.03 ± 3.29	2.69 ± 1.62	0.73 ± 1.42	**12.39**
841	2-Methylbutanoic acid*		0.20 ± 0.71	1.31 ± 1.83	4.72
849^d^	Ethyl 2-methylbutanoate			0.08 ± 0.18	1.34
854	(*Z*)-3-Hexen-1-ol*	76.98 ± 88.11	10.03 ± 8.97	34.15 ± 39.66	−3.52
867	1-Hexanol*	1.80 ± 3.11	0.90 ± 1.03	1.37 ± 1.63	−1.39
943	3-Octanone*	4.67 ± 2.03	16.92 ± 11.73	33.87 ± 35.12	6.20
986^e^	4-lsothiocyanato-1-butene	14.84 ± 12.92	12.06 ± 12.19	21.06 ± 20.03	−0.39
998	Ethyl hexanoate*		0.44 ± 0.80	0.59 ± 1.14	−3.04
1005	(*Z*)-3-Hexenyl acetate*	83.83 ± 72.60	22.55 ± 19.00	16.73 ± 24.49	2.79
Aromatic compounds					
963	Benzaldehyde*	25.61 ± 17.98	10.96 ± 9.61	6.08 ± 3.65	7.03
1038	Benzyl alcohol*	2.96 ± 0.90	7.03 ± 22.72	1.37 ± 1.13	1.93
1101	Methyl benzonate*	1.09 ± 1.88			0.00
1118	2-Phenylethyl alcohol*		0.05 ± 0.22	0.34 ± 0.95	−1.66
1145	Phenylacetonitrile*	0.61 ± 1.06			0.00
1169	Benzyl acetate*	0.24 ± 0.22	0.37 ± 0.73	0.33 ± 0.31	−1.37
1300	Indole*	2.24 ± 3.88			0.00
Terpenoids					
1099	Linalool*	0.49 ± 0.86	3.12 ± 6.57	0.34 ± 0.65	1.48
1432^f^	Longifolene	2.24 ± 1.01	1.33 ± 0.94	0.42 ± 0.33	**23.72**
Unknowns					
805	m/z:45,57,58,43,59,41		0.65 ± 2.40	3.68 ± 2.84	**20.42**
936	m/z:115.41.56.57.86.39		1.08 ± 1.77	2.73 ± 3.48	2.63
1088	m/z:41,70,67,126,127,99		0.51 ± 0.77	0.35 ± 0.61	−2.73
1130	m/z:113,119,45,134,73,53	8.13 ± 7.96	1.84 ± 4.51	0.33 ± 0.55	3.48
1217	m/z:56,41,114,55,70,42		0.64 ± 0.66		**18.13**
1228	m/z:56,55,41,114,70,42		1.50 ± 0.94		**45.86**

^a^RI 717, Cajka et al. 2007, J Sep Sci 30:534–546; ^b^ RI 732, Sebastian et al. 2003, Sci des Alim 23:497–511; ^c^ RI 827, King et al. 1993, J Agric Food Chem 41:1974–1981; ^d^ RI 849, Mantzouridou et al. 2006, J Agric Food Chem 54:2695–2704; ^e^ RI 984, Bergnaud et al. 2003, Int J Environ Anal Chem 83:837–849; ^f^ RI 1416, Kant et al. 2004, Plant Physiol 135:483–495

In conclusion, our results clearly demonstrate the involvement of bacteria in shaping the scent emission of flowers. Bacterial contributions to the formation of VOCs that are perceived by both pollinators and antagonistic organisms demand a rethinking about the ecology and evolution of scent-mediated interactions between flowers and other organisms. Floral scents are crucial in initiating and maintaining flower constancy, which is prerequisite for effective pollen transfer between conspecific flowers (Borghi et al. 2017). Given that bacteria reduce, alter, or emit behaviorally relevant VOCs, these modifications of floral scent may prevent initial attraction either by reducing the emission rate of attractive compounds or by adding repellent compounds to the bouquet. Furthermore, associative learning may be hampered if scent bouquets differ between flowers due to differences in bacterial colonizers. The number of studies on possible ecological consequences of bacterial contributions to the floral phenotype is limited but our results underline that bacteria should not be overlooked when studying floral scents and the interactions they mediate.
